# Hexacene on Cu(110) and Ag(110): Influence of the Substrate on Molecular Orientation and Interfacial Charge Transfer

**DOI:** 10.1021/acs.jpcc.2c00081

**Published:** 2022-03-07

**Authors:** Marie S. Sättele, Andreas Windischbacher, Katharina Greulich, Larissa Egger, Anja Haags, Hans Kirschner, Ruslan Ovsyannikov, Erika Giangrisostomi, Alexander Gottwald, Mathias Richter, Serguei Soubatch, F. Stefan Tautz, Michael G. Ramsey, Peter Puschnig, Georg Koller, Holger F. Bettinger, Thomas Chassé, Heiko Peisert

**Affiliations:** † Institute of Physical and Theoretical Chemistry, University of Tübingen, Auf der Morgenstelle 18, 72076 Tübingen, Germany; ‡ Institute of Organic Chemistry, University of Tübingen, Auf der Morgenstelle 18, 72076 Tübingen, Germany; § Institute of Physics, University of Graz, NAWI Graz, Universitätsplatz 5, 8010 Graz, Austria; ∥ Peter Grünberg Institut (PGI-3), Forschungszentrum Jülich, 52425 Jülich, Germany; ⊥ Jülich Aachen Research Alliance (JARA), Fundamentals of Future Information Technology, 52425 Jülich, Germany; # Experimental Physics IV A, RWTH Aachen University, 52074 Aachen, Germany; ○ Physikalisch-Technische Bundesanstalt, Abbestrasse 2-12, 10587 Berlin, Germany; ¶ Institute for Methods and Instrumentation in Synchrotron Radiation Research, Helmholtz-Zentrum Berlin für Materialien und Energie GmbH, Albert-Einstein-Straße 15, 12489 Berlin, Germany; ▽ Center for Light-Matter Interaction, Sensors & Analytics (LISA+), University of Tübingen, Auf der Morgenstelle 18, 72076 Tübingen, Germany

## Abstract

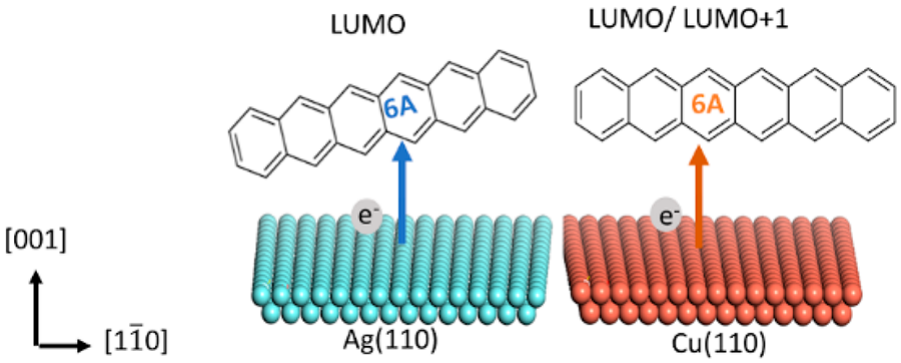

Hexacene, composed of six linearly fused benzene rings, is an organic semiconductor material with superior electronic properties. The fundamental understanding of the electronic and chemical properties is prerequisite to any possible application in devices. We investigate the orientation and interface properties of highly ordered hexacene monolayers on Ag(110) and Cu(110) with X-ray photoemission spectroscopy (XPS), photoemission orbital tomography (POT), X-ray absorption spectroscopy (XAS), low-energy electron diffraction (LEED), scanning tunneling microscopy (STM), and density functional theory (DFT). We find pronounced differences in the structural arrangement of the molecules and the electronic properties at the metal/organic interfaces for the two substrates. While on Cu(110) the molecules adsorb with their long molecular axis parallel to the high symmetry substrate direction, on Ag(110), hexacene adsorbs in an azimuthally slightly rotated geometry with respect to the metal rows of the substrate. In both cases, molecular planes are oriented parallel to the substrate. A pronounced charge transfer from both substrates to different molecular states affects the effective charge of different C atoms of the molecule. Through analysis of experimental and theoretical data, we found out that on Ag(110) the LUMO of the molecule is occupied through charge transfer from the metal, whereas on Cu(110) even the LUMO+1 receives a charge. Interface dipoles are determined to a large extent by the push-back effect, which are also found to differ significantly between **6A**/Ag(110) and **6A**/Cu(110).

## 1 Introduction

Organic π-conjugated molecules have gained a major role in the development of modern electronic technologies. A promising group of organic semiconductor materials are the homologous series of acenes, which consist of several linearly fused benzene rings.^1−3^ With increasing size of the π-system, the energetic distance between highest occupied molecular orbital (HOMO) and lowest unoccupied orbital (LUMO) as well as the reorganization energy decreases,^4−6^ while the charge carrier mobility typically increases.^7^ Therefore, larger acenes beyond pentacene (**5A**) are promising candidates for applications in optoelectronic devices.^8,9^ Due to their pronounced instability toward light and oxygen, large acenes are difficult to handle under normal conditions.^10^ Despite this, the formation of comparably stable, ordered film structures was recently reported for hexacene (**6A**) and heptacene (**7A**).^3,8,11−13^ Additionally, in particular on-surface synthesis opened an entrance to larger acenes up to dodecacene.^14−20^

The interaction of π-conjugated molecules and possible contacts like the coinage metal surfaces of copper and silver is known to change the electronic structure distinctly because of charge transfer and chemisorption.^21−24^ Furthermore, for heptacene (**7A**) on Cu(110), it was demonstrated that even the adsorption geometry affects the interfacial electronic structure.^13^

Systems where the structural ordering of the first monolayers have been studied comprehensively comprise tetracene (**4A**) and pentacene (**5A**) on Ag(110) and Cu(110).^25−31^ Generally, the adsorption geometry depend crucially on both, the intermolecular and molecule–substrate interactions. For **4A** on Ag(110) various adsorption geometries were observed as a function of the coverage in the (sub)monolayer range, stabilized by different intermolecular interactions (head-to-head, corner-to-corner, and side-by-side).^27^ For a coverage of a saturated monolayer of **4A**, the molecules are rotated by ±10° with respect to the [11̅0]-direction of the anisotropic substrate surface.^27^ In contrast, single domains were observed for a monolayer of **5A** on Ag(110), in which the long molecular axis is oriented perpendicular to the [11̅0]-direction of the substrate.^24^ We note that multiple monolayer phases of **5A** have been discussed widely in the literature and shown to depend on parameters like coverage, annealing temperature, and evaporation rate (for **5A** on Cu(110), see, e.g., refs (25 and 28)). The direct comparison of **5A** on Ag(110) and Cu(110) prepared under same conditions shows that also the substrate affects the adsorption geometry significantly.^24^ These examples emphasize that the adsorption geometry may crucially depend on both the length of the acene and/or details of the preparation. Recently, it was demonstrated for heptacene monolayers on Cu(110) that the orientation of the adsorbate may be even crucial for the charge transfer processes.^13^

In light of this, we present a combined study of the adsorption geometry and interfacial electronic structure of **6A** monolayers on Cu(110) and Ag(110) prepared under identical conditions. We apply complementary surface science methods including X-ray photoemission spectrocopy (XPS), photoemission orbital tomography (POT), scanning tunneling microscopy (STM), X-ray absorption spectroscopy (XAS), and low-energy electron diffraction (LEED). Our experiments are flanked by density functional theory (DFT) calculations. Comparing the results with other acenes, we offer a comprehensive description of the electronic structure of the molecule on coinage metals.

## 2 Methods

The sample preparation for all experiments were conducted in ultrahigh vacuum chambers. The Ag(110) and Cu(110) substrates were cleaned by repeating cycles of Ar^+^ ion sputtering (15 min, 0.8 kV) and annealing (20 min, 500 °C). **6A** was synthesized according to the literature,^11^ sublimed in vacuum from a Knudsen type evaporator at a temperature of 270 °C and adsorbed on the metal substrates held at room temperature.

The STM and LEED measurements were carried out in a two-chamber UHV vessel equipped with a low-energy electron diffraction (LEED) unit from OCI Vacuum Microengineering Inc. and a variable-temperature (VT)-STM from Omicron GmbH. The STM measurements were performed with a mechanically cut Pt/Ir tip. The tunneling voltages are referenced to the sample. The WSxM^32^ and LEEDpat^33^ programs were used for analysis of, respectively, STM and LEED data.

XPS and UPS measurements were carried out in a multichamber UHV system at a typical base pressure of 3 × 10^–10^ mbar. The analysis chamber is equipped with a Phoibos 150 hemispherical electron analyzer (SPECS), a monochromated X-ray source (XR 50 M, SPECS), an ultraviolet light source (UVS 300, SPECS) and an Omicron LEED system. The XPS measurements were performed with an energy resolution of 400 meV, measured with the width of the Fermi edge. Monochromatic Al K_α_ radiation (1486.74 eV) was used. The angle between analyzer and X-ray source was 54°. XPS spectra were evaluated and fitted with the program Unifit 2018.^34^ The relative intensities of C 1s, Ag 3d, and Cu 2p lines were used to determine the thickness of the molecular layers assuming the Frank–van der Merwe growth mechanism of the latter. We used sensitivity factors from Yeh and Lindau^35^ and mean free paths for organic molecules calculated according to Seah and Dench.^36^ Taking into account the molecule–molecule distance reported for grown pentacene crystals^37^ and the assumption of flat-lying molecules, we estimated the thickness of a saturated monolayer to be 0.4 nm.

The X-ray absorption spectroscopy (XAS) experiments were performed at the PM4 beamline of the BESSY II electron storage ring operated by the Helmholtz-Zentrum Berlin (HZB) using the low-dose endstation.^38^ The absorption was measured indirectly by detecting the total electron yield (sample drain current). The energy resolution was about 100 meV for a photon energy of 285 eV. For the angle-resolved measurements, the sample was rotated along the [11̅0]-direction while keeping the azimuthal orientation of the p-polarized light fixed. The XAS spectra were normalized by the sample height well above the ionization threshold.

The electronic properties of **6A**/Ag(110) and **6A**/Cu(110) were calculated within the framework of density functional theory (DFT). For the simulation of periodic interfaces, we utilized the GPAW^39−41^ code (version 21.1.0). Exchange-correlation effects were approximated by the functional of Perdew–Burke–Ernzerhof (PBE)^42^ and van der Waals contributions were treated with Grimme’s D3 dispersion correction.^43^ We used the projector-augmented wave (PAW) method^44^ assuming an energy cutoff of 400 eV. The ionic positions of all optimized molecules were calculated until the remaining forces were below 0.01 eV/Å applying a Gaussian smearing of 0.01 eV. We adapted the experimentally determined unit cells from the LEED experiments and simulated the surface using five metallic layers and a 30 Å vacuum layer within the repeated slab approach. To prevent disturbing spurious electrical fields, a dipole layer was placed in the vacuum region.^45^ We used a Monkhorst–Pack^46^ 6 × 2 × 1 grid of *k-*points constraining the coordinates of the two bottom Cu and Ag layers of the slab for the structure optimization. The XPS binding energies were calculated on the same level of theory using the delta Kohn–Sham total energy differences method, in which the energies of the C 1s core level excitations are determined as the total energy differences between the ground state and the first core ionized states.^41^ For the ionized states, the core electrons of each target atom were modeled by a C 1s core-hole setup, while a charge was reintroduced at the Fermi level to ensure neutrality of the periodic unit cell. While the Kohn–Sham procedure should give consistent results for all atoms of the same kind, the absolute binding energies depend on the exchange-correlation functional. Therefore, the calculated energy scale was rigidly shifted to align with experiment.

POT measurements were performed at the insertion device beamline of the Metrology Light Source of the Physikalisch-Technische Bundesanstalt (Berlin, Germany). We used the p-polarized light of 35 eV photon energy in the 40° incidence geometry with respect to the surface normal. The emitted photoelectrons were collected in a broad (±80°) angle range and analyzed in angle- and energy-resolved manner by the toroidal electron analyzer.^47^ The maps of photoemission distribution in momentum space at chosen binding energies were obtained by rotating the sample around its normal in 2° steps.

To simulate the POT momentum maps, we recalculated the Kohn–Sham energies and wave functions of **6A**/Ag(110) and **6A**/Cu(110) non-self-consistently with the Vienna Ab Initio Simulation Package (VASP), version 5.4.4.^48,49^ The *k*-point mesh of 12 × 5 × 3 was used for simulations. The one-step model of photoemission^50^ was utilized to simulate the angle-resolved photoemission momentum maps under assumption that the wave function of the final state can be described as a plane wave.^24^ The simulations were corrected by an exponential damping factor, which takes the mean free path of the photoemitted electrons into account.^51^

Quantum chemical calculations of isolated molecules were performed with the ORCA package.^52^ The geometry was optimized with the global hybrid B3LYP functional^53,54^ in combination with the def2-TZVP basis set.^55^ The K-edge XAS spectra were simulated by applying time-dependent density functional theory as implemented in ORCA:^56^ Symmetry-equivalent C 1s orbitals were localized using the Pipek–Mezey procedure,^57^ and the TDDFT calculations were carried out by allowing only excitations from the localized 1s orbitals. For a better comparison with the experimental spectra, the discrete excitations were broadened with Gaussian functions of increasing width as described in ref (58).

## 3 Results and Discussion

### 3.1 Arrangement of **6A** Molecules in Monolayer Structures

Before we focus on the electronic structures of the interfaces, we characterize the molecular arrangements on the surfaces. For this, we rely on STM and LEED measurements of monolayers of **6A** prepared either by thermal evaporation to give a full monolayer on the metal substrates held at room temperature or, alternatively, by deposition of a multilayer and subsequent annealing at 270 °C for 1 min. We note that both approaches result in the same adsorption geometry (Supporting Information, Figure S1).

**Figure 1:**
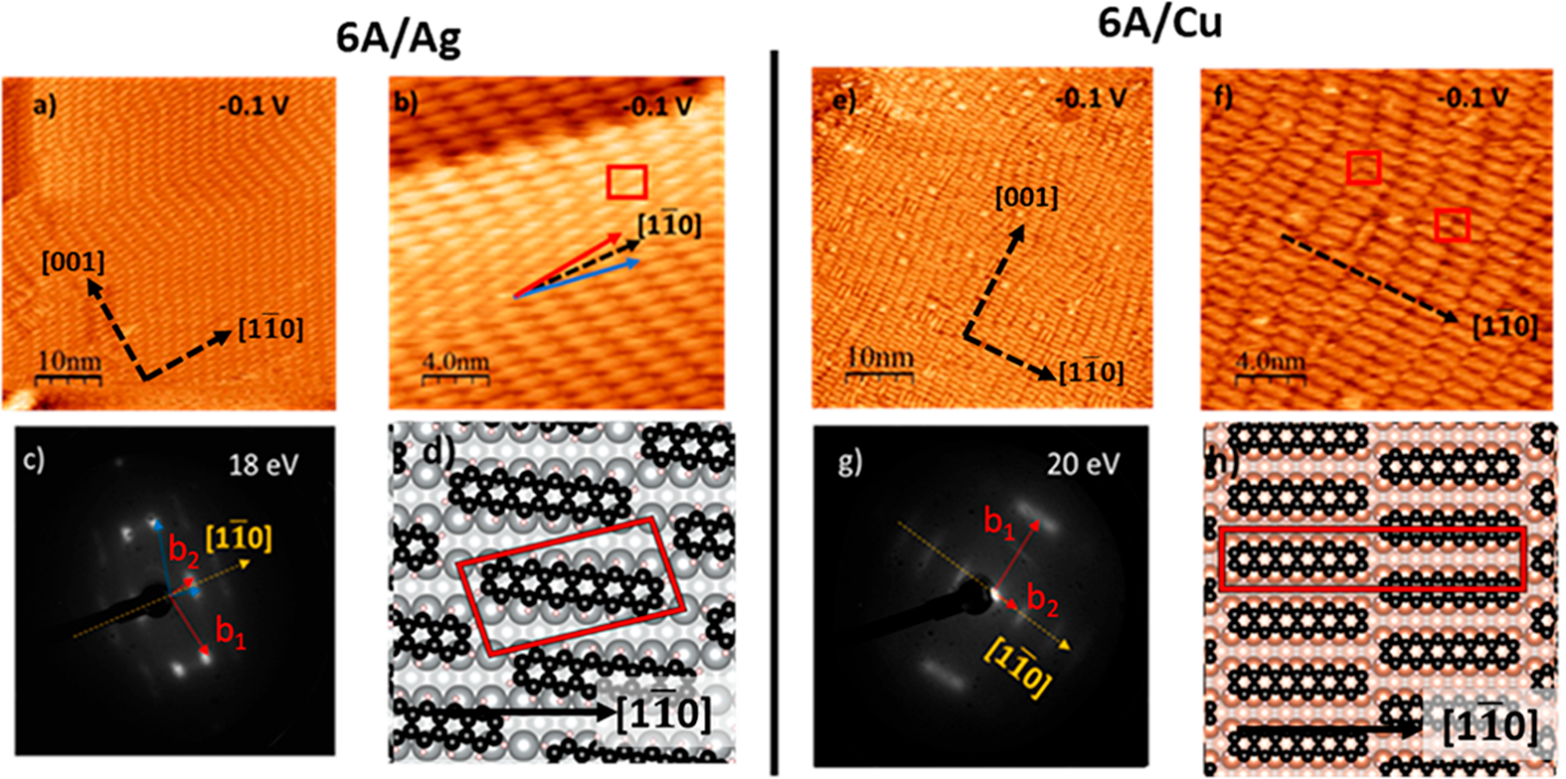
(a–d) Saturated monolayer of **6A** on Ag(110): (a, b) overview and zoomed-in STM images measured at, respectively, *I* = −600 pA, *V* = −0.1 V and *I* = −300 pA, *V* = −0.1 V, (c) LEED pattern measured at 18 eV and (d) the structural model. (e–h) Saturated monolayer of **6A** on Cu(110): (e, f) overview and zoomed-in STM images measured at *I* = −300 pA, *V* = −0.1 V, (g) LEED pattern measured at 20 eV, and (h) the structural model of the staggered molecular arrangement. Note that in part g, the sample was slightly tilted to show the (0, 0) spot.

In Figure 1, we show STM images of **6A** monolayers on Ag (a–d) and Cu surfaces (e–h). For both substrates, STM images revel a high degree of ordering. On both surfaces the molecules are essentially oriented close to the metal [11̅0]-rows with few defects. However, there are some noteworthy differences. On Ag(110), we observe two different domains, where the orientation of the long molecular axis is misaligned by ±6° with respect to the [11̅0]-direction of the substrate (Figure 1b). For the Cu interface, STM images reveal that most molecules are also oriented along the [11̅0]-substrate direction (parallel to closed-packed Cu rows), but a minority of molecules are along [001], i.e., 90° rotated. In contrast to Ag, for **6A** on Cu no rotational misalignment is apparent. This may be due to the high commensurability of **6A** and Cu. The surface unit cell distance of Cu along the [11̅0]-direction (Cu: 2.56 Å) shows good agreement with the width of a benzene-ring (2.45 Å), while this is not the case for Ag (Ag: 2.89 Å). Such a good match may support the preferred [11̅0]-orientation of the molecules in the direction of the Cu rows.

To determine the surface unit cells of the two above monolayers, we recorded LEED images (Figure 1c,g). At the low incident electron beam energy of 20 eV, the LEED pattern are mainly related to the molecular unit cell. LEED images at higher energies were used to determine the size and orientation of the unit cell of the adlayer with respect to the crystal directions more exactly (Figure S4, Supporting Information). On Ag, the LEED pattern indicates the presence of a long-range ordering within the monolayer and can be assigned to two different lattices, mirrored at the [11̅0]-direction of the Ag(110) surface (Figure 1c, the reciprocal vectors are marked by blue and red arrows). The similar brightness of the pattern indicates that both domains are evenly distributed. The two domains can be described in matrix notations by  and  (analyzed with LEEDpat^33^).

On Cu, the LEED pattern suggests similar unit cell dimensions as on Ag, however, a definite assignment to a superstructure is difficult due to broadened diffraction spots. The blurred spots for the Cu(110) examples seems to be due to a small coherence length. STM proofs that the size of homogeneously ordered domains is comparably short compared to Ag(110), as the molecular rows along [001] show a wave-like structure. Along [11̅0], we find both, a staggered arrangement of the molecules (corner-to-corner) and an orientation in which the ends of the molecules are arranged directly one behind the other (head-by-head), highlighted by red squares in Figure 1f. The reciprocal lattice vectors of a possible *c*(14 × 2) superstructure are indicated by red arrows in Figure 1g.

Real space models based on the findings of our STM and LEED investigations are shown in Figure 1, parts d and h. These models were used for the DFT simulations of **6A** on Ag(110) and Cu(110). Compared to its neighbors in the acene series, the unit cell for **6A** on Ag(110) is reminiscent of the reported superstructure of **4A** on Ag(110): .^27^ Moreover, we highlight that we also observed a mirror-symmetric  unit cell for **7A** on Ag(110) under similar preparation conditions (see Supporting Information, Figures S2 and S3). Apparently, only the length of the superstructure vector **b**_**2**_ in the direction of the substrate vector **a**_**2**_ is varied (4·**a**_**2**_ for **4A**, 6·**a**_**2**_ for **6A** and 7·**a**_**2**_ for **7A**). Thus, it seems that only the length of the acene affects the slightly different geometry and the misalignment on Ag(110). Albeit that such domains mirrored at the [11̅0]-direction of the Ag(110) surface have not been observed for **5A** on Ag(110) yet, we cannot rule out that they can be formed under certain preparation conditions. On Cu, there is a clearly preferred orientation along the [11̅0] rows; however, with some molecules rotated by 90°, we see an indication for a partial reorientation, which is not reported for **4A** or **5A**. This tendency to reorient has been shown to be even more pronounced for **7A** leading to the observed temperature dependent phases of **7A**/Cu(110).^13^

### 3.2 Electronic Structure of **6A** Molecules on Ag(110) and Cu(110)

The question may arise whether the type of substrate or the molecular orientation affects the interactions at the interface more strongly.

We start by probing the valence region of the molecules using ultraviolet photoemission spectroscopy (UPS). The obtained valence band spectra (Supporting Information Figure S5) show distinct differences between monolayers of **6A** on both surfaces, in particular peak positions at 0.2, 0.9, 1.9, and 2.9 eV on Ag and 0.2 and 1 eV on Cu. We can assign those peaks from Figure S5 to emissions from molecular orbitals utilizing photoemission orbital tomography (POT), which combines angle-resolved UPS (ARUPS) measurements with density functional theory calculations (DFT). POT has already been applied successfully to explain several acene/coinage metal systems.^3,13,24,59^

**Figure 2:**
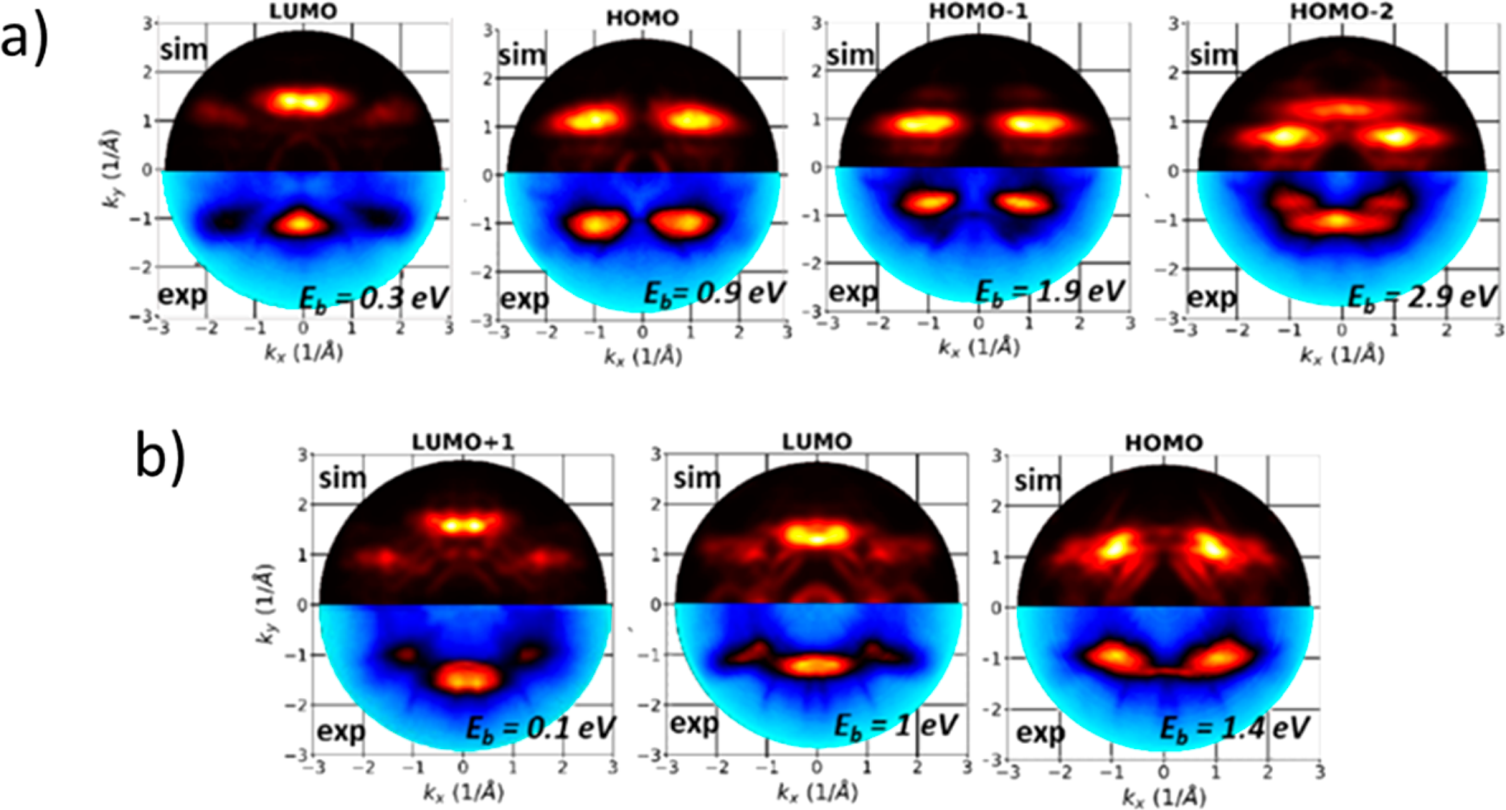
Experimental momentum maps (lower halves) compared to calculated momentum maps (upper halves) of the **6A**/Ag (110) (a) and **6A**/Cu(110) interfaces (b).

Experimental momentum maps measured at different binding energies are compared to calculated momentum maps of the molecules at the different interfaces in Figure 2. The calculated maps of the isolated molecule are shown as a reference in Figure S6 in the Supporting Information. For **6A** on Ag(110) (Figure 2a), the comparison allows the identification of four molecular emissions, namely LUMO, HOMO, HOMO–1, and HOMO–2. The maps of HOMO, HOMO–1, and HOMO–2 can be clearly differentiated as their intensity maxima appear at different binding energies (increasing from 2.9 to 0.9 eV) and different *k*-values (increasing from 0.8 to 1.3 Å^–1^). Also for **6A**/Cu(110), the experimental maps are in an almost perfect agreement with the simulations of the molecular monolayer on the surface (Figure 2b). We can assign the emission pattern to the LUMO+1, LUMO, and HOMO of the **6A** molecule. The LUMO and LUMO+1 can be distinguished in their momentum maps due to differences in the *k*_*y*_-values of their main lobes along *k*_*x*_ (1.6 vs 1.8 Å^–1^).

Analysis of our POT results complements our structure analysis, as it confirms the orientation of both molecules along the [11̅0] direction. The considerable structural disorder that is apparent in Figure 1, parts a and b, makes the rotation of ±6° on Ag(110) undetectable. Moreover, POT shows that charge transfer is present upon absorption of **6A** in both systems; however, our POT results point at a significant difference: while the LUMO of **6A** gets occupied in both cases, on Cu, also the LUMO+1 receives charge.

With UPS, we also obtain the work functions of both interfaces in focus.^13,60^ Molecules close to a metal interface tend to have a reduced HOMO–LUMO gap by the polarization of the metal.^61−64^ Consequently the LUMO level moves closer to the Fermi level *E*_F_.

**Figure 3:**
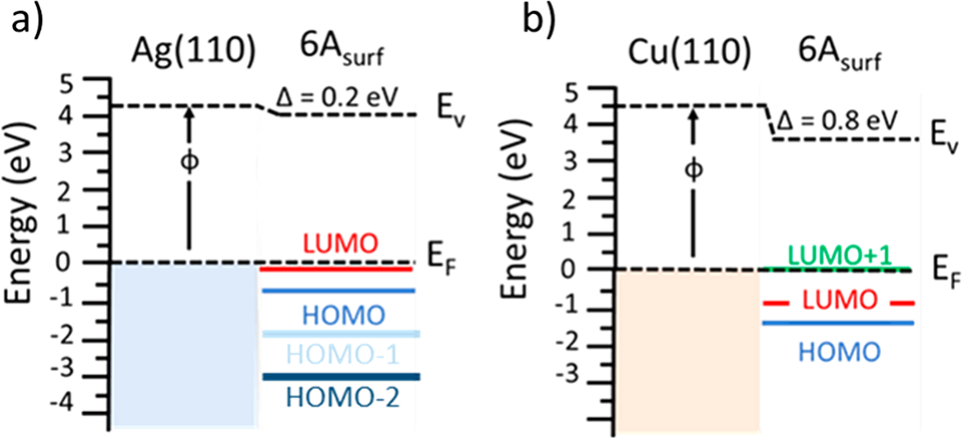
Energy level alignment of **6A** monolayers on Ag(110) (a) and Cu(110) (b). We used experimentally determined values (from UPS and POT) for work functions and energy levels to describe the interfaces. These agree well with the calculated energy level alignments (cf. Figure S9 in the Supporting Information).

Finally, using the combined results of photoemission study, namely after assignment of observed molecular emissions to particular molecular orbitals and obtaining the work function values, we can describe details of the energy level alignment of **6A**/Ag(110) and **6A**/Cu(110) schematically shown in Figure 3, parts a and b. Upon adsorption, complex redistributions of electrons at the interfaces contribute to a change of surface dipoles, and consequently, to a change of the work function (also compare the calculated charge density differences in Figures S7 and S8 in the Supporting Information).

Here, pushing the electron tail of the metal surface back to the surface, leads to a reduction of the work function of the substrate (i.e., Pauli repulsion, push-back effect, e.g. refs (60 and 65)). Conversely, charge transfer from the substrate to the molecules leads to an increase of the work function at the interface. As can be seen from Figure 3, we measured a reduction of the work function *via* UPS of 0.2 and 0.8 eV for **6A**/Ag(110) and **6A**/Cu110), as a consequence of the adsorption. Thus, an interface dipole has been formed. Due to the charge transfer, the occupied LUMO or LUMO+1 is pinned close the Fermi level, causing a down-shift of the HOMO level. Apparently, and contrary to expectations, the work function for the interface with the larger charge transfer to the LUMO+1 state (**6A** on Cu(110)) is reduced more strongly. This can be understood, if the effect of the charge transfer is differently compensated by the push-back effect.

In Table 1, we thus analyze various contributions to the total work function change, Δϕ_sim_. With the help of DFT calculations, we approximate the dipole arising from the bend of the molecule, Δϕ_bend_ the charge transfer, Δϕ_CT_ and the electron push-back, Δϕ_push-back_. Note that, due to the theoretical approximations, the calculated factors should not be taken as absolute values. The results of such an analysis represent qualitative numbers in order to better understand the interfaces and the experimental trends.

**Table 1:** Experimentally Determined Work Function Changes Δϕ_exp_ and Calculated Work Function Changes, Δϕ_sim_ as Obtained from PBE+D3 Calculations for the Hollow Adsorption Configuration with 0° and 6° Rotation of the Long Molecular Axis out of the [11̅0]-Direction for **6A** on Cu(110) and Ag(110) and Decomposition of the Calculated Work Function Change in Δϕ_bend_, Δϕ_CT_, and ϕ_push-back_

	6A/Cu	**6A**/Ag
Δϕ_exp_ [eV] change	–0.8	–0.2
Δϕ_sim_ [eV]	–0.88	–0.16
Δϕ_CT_ [eV]	0.73	0.36
Δϕ_push-back_ [eV]	–1.28	–0.40
Δϕ_bend_ [eV]	–0.33	–0.12

Specifically, the total work function change Δϕ_sim_ was described considering the following different factors:

- A distortion of the geometry of the molecules upon adsorption, i.e., a bend of the planar **6A** toward the surface, leading to an internal dipole of the molecule Δϕ_bend_. This change is calculated as the vacuum potential step of a freestanding monolayer of the molecule in its already distorted geometry.
- Electron transfer from the metal to the molecules (Δϕ_CT_). The transferred electrons are measured via Bader charge analysis.^66^ Subsequently, the influence on the work function is calculated employing a simple capacitor model.^67^
- Push-back of electrons into the substrate upon adsorption of an organic molecule Δϕ_push-back_, which is assumed to be the remaining contribution to the totally calculated work function change Δϕ_tot_.

The calculated work function changes reproduce the experimental results very well. Small deviations might be caused by subtle differences in the monolayer structures in ideal theory and experiment as well as the chosen exchange-correlation functional. On Cu(110), the dipole caused by charge transfer is overcompensated essentially by the opposite, very large dipole arising from the push-back effect. On Ag(110), the interaction with the substrate and, therefore, also the influence of the push-back is smaller. In case of a strong interaction between molecule and metal surface, the interface dipole is apparently not a direct measure for the charge transfer at the interface.

The different bonding situation is in line with the calculated molecular adsorption heights of hexacene, i.e.: ∼2.2 and ∼2.6 Å on Cu and Ag, respectively (Figures S7 and S8, Supporting Information). Estimating the limit for physisorption by the sum of the van der Waals radii (Cu, 1.4 Å; Ag, 1.72 Å; C, 1.7 Å) of different metals and the molecule (on Cu, 3.1 Å; on Ag, 3.42 Å), we may conclude about chemisorption for both systems. The influence of the substrate on the bonding of acenes can also be compared to the shorter acenes. This further reflects in the average vertical substrate-molecule distance, which can be determined, e.g., by X-ray-standing wave measurements.^68^ For a 0.7 ML **5A** layer on Ag(111) at room temperature, the adsorption height of the molecules (3.12 Å)^69^ is significantly larger than on Cu(111) (2.34 Å).^23^ UPS measurements find that the LUMO is only fractionally filled for **5A** on Ag(110), while on Cu(110) the LUMO is fully occupied.^24^ A reason for the short adsorption distance might be the strong organic/metal chemisorption involving a hybridization of molecular orbitals and metal states.^21,24,70^ This finding is likely supported by the very good structural fit of the acene repeat unit with the lattice spacings of the Cu(110) surface. However, the LUMO+1 is never involved in the interfacial charge transfer in those cases. Compared with **5A**, we do expect an even stronger bond between metal and **6A** molecules due to the increased electron affinity.^71^ This goes hand in hand with Clar’s π-sextet rule,^72,73^ according to which the stability of larger acenes decreases rapidly with increasing number of benzene-rings pointing toward their higher reactivity.

**Figure 4:**
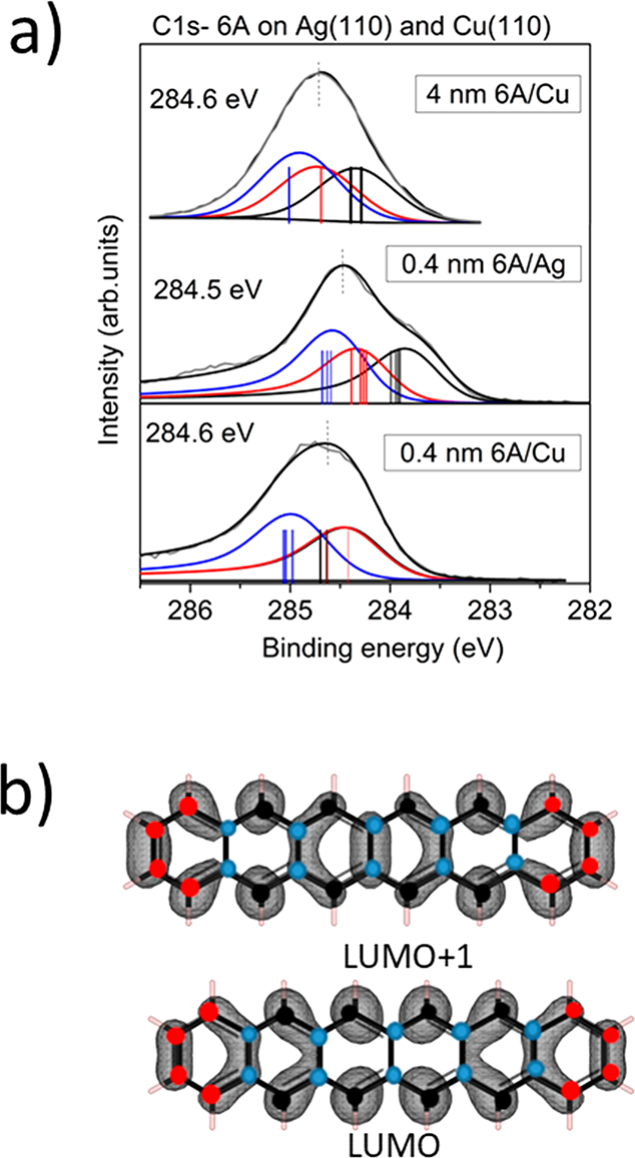
(a) Experimental C 1s core level spectra of **6A** monolayer (nominal coverage of 0.4 nm) on Cu(110) (middle) and Ag(110) (bottom) fitted by three different components compared to that of a 4 nm thick multilayer (top). The three components can be attributed to the carbon atoms labeled by different colors in part b. Bars are related to binding energies of manifold carbon atoms of the isolated **6A** molecule (top) and **6A** at the interfaces (middle, bottom) as obtained from GPAW. (b) Real-space representations of LUMO+1 and LUMO of the isolated **6A** molecule calculated with GPAW.

In the previous section, we showed that a significant charge transfer occurs between the metal substrates and **6A**. By measuring X-ray photoemission spectroscopy (XPS), we demonstrate now the effect of this metal-molecule interaction on the core electrons of the molecule. In Figure 4a, C 1s core level spectra of a 4 nm thick film is compared to the spectra of **6A** monolayers on Ag(110) and Cu(110). The C 1s peak shape of the thick **6A** film is in a good agreement with recently published data of **6A** films on Cu(110)-O(2×1)^12^ and Au(110).^11^ Calculated core level binding energies for each carbon atom according to delta Kohn–Sham calculations are included as colored bars in Figure 4a; the colors are related to different carbon atoms as indicated in Figure 4b.

For the isolated **6A** molecule, the calculations suggest that three chemically different (i.e., in different chemical environments) carbon species can be distinguished: inner C–C (blue), outer C–H (black), and the terminal C–H bonded atoms (red). Indeed, the spectrum of the 4 nm film in Figure 4a can be well described using these three components in their stoichiometric ratio. The relative ordering of these components in Figure 4a is based on their calculated binding energies [bars in Figure 4] and in agreement with the literature.^74^ Based on the calculations, we assigned that the inner C–C appear at the highest binding energy, followed by the terminal C–H and inner C–H. Peak fit parameters are summarized in Tables S1–S3 (Supporting Information).

The shapes of the monolayer spectra in Figure 4 are distinctly different from those in the spectrum of the thick film: Intensity at the low-binding energy side develops, and a tail at the high-binding energy side is visible. The asymmetric C 1s spectra on both substrates tails toward higher binding energies, described by an asymmetric Doniach–Sunjic profile in the peak fits, and it indicates a strong coupling of **6A** molecules to both metallic substrates.

For **6A** monolayers on Ag(110), the whole XPS spectrum shifts to lower binding energies compared to the bulk. The overall shift of the spectrum to lower binding energies can be explained by the observed charge transfer from the metal to the **6A** molecule. However, final state screening effects in photoemission at the interface to metal substrates cause also a lowering of binding energies (e.g., refs (75 and 76)). In addition, the energy level alignment at the interface may affect absolute core level binding energies distinctly, e.g., due to a pinning at the LUMO or LUMO+1.

However, not only an overall shift of the C 1s binding energy is observed for **6A** on Ag(110) in Figure 4a but also a change of the peak shape that is caused by a relative shift of the different components. On Ag(110), the LUMO of **6A** is filled due to the charge transfer from the substrate to the molecule. The electron density of the LUMO is mainly located at the inner C–H (black) with less contributions on the terminal C–H (red, compare Figure 4b). In the case of a local charge transfer to certain carbon atoms at the interface, an energetic shift of the respective component toward a lower binding energy would be expected. Indeed, we observe a stronger shift for these components leading to the visible shoulder of the spectrum at lower binding energies.

For **6A** on Cu(110), the electron transfer from the metal into **6A** is even more pronounced, and so the LUMO+1 becomes gradually filled. As a consequence, the higher binding energy of the C 1s spectrum compared to that of **6A** on Ag(110) is most likely caused by the different energy level alignment at the interface (pinning at the LUMO+1). We also note that the inner and terminal C–H atoms (black and red) now appear at the same binding energy according to the experimental fit as well as in the calculations. This may be rationalized by the adsorption geometry, where both atom species are located at the bridge position along the Cu rows (Figures S7 and S8, Supporting Information). Therefore, their chemical environment becomes more similar. Thus, the two different C 1s peak shapes of the monolayer spectra on Ag(110) and Cu(110) reveal a strong influence of geometric and electronic effects, leading to different energetic shifts of the nonequivalent carbon atoms.

Finally, we probe the electron transition from core levels, here the C 1s, to unoccupied molecular states, such as LUMO and LUMO+1, using XAS. This method, thus, directly provides complementary results to our POT measurements, i.e., information on the charge transfer from the substrate to the unoccupied molecular states.

**Figure 5:**
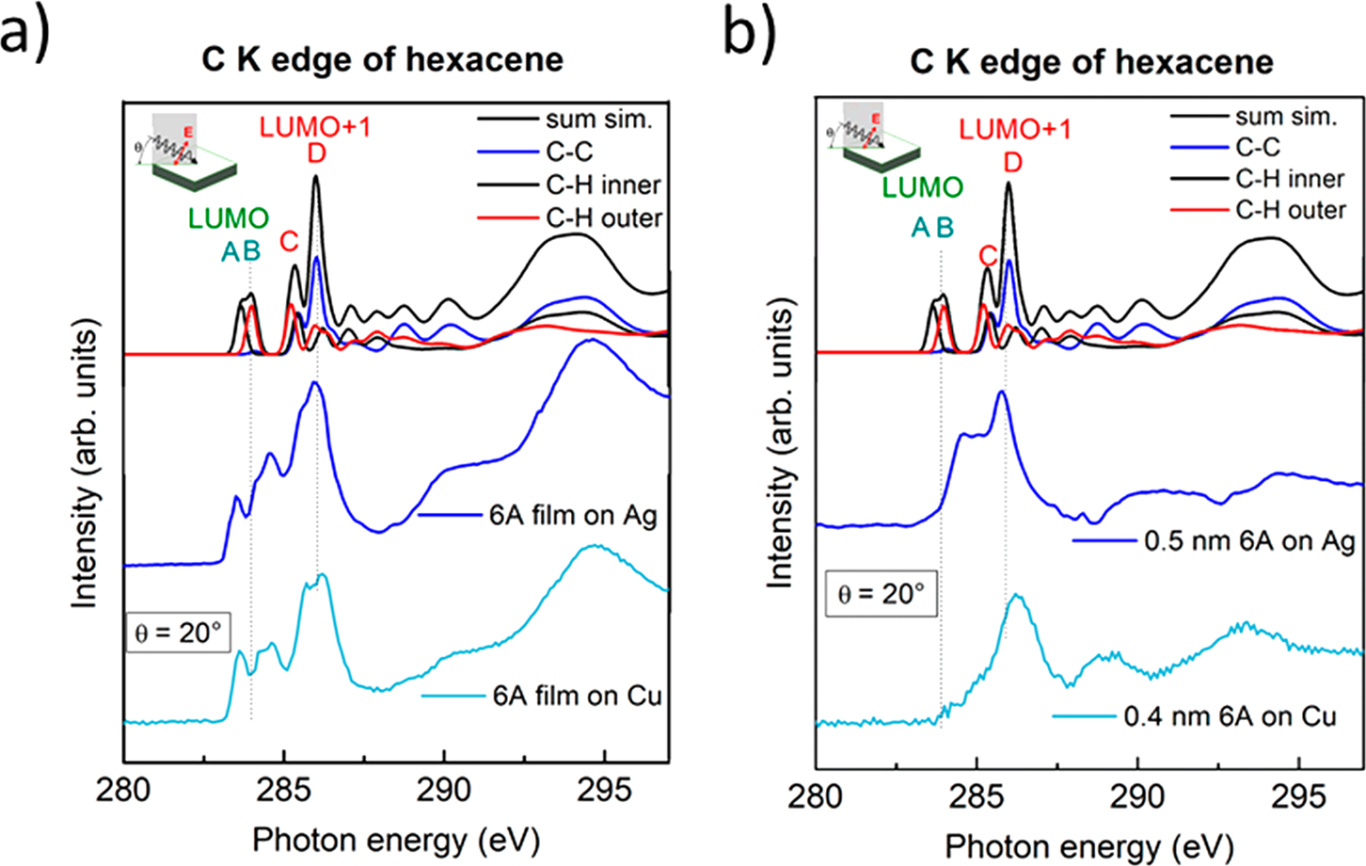
Simulated and experimental C K edge XAS spectra of **6A**. The B3LYP/def2-TZP level of theory was used for calculation. (a) Thick films (9 and 3.6 nm for Cu(110) and Ag(110), respectively) and (b) monolayers (0.4 and 0.5 nm for Cu(110) and Ag(110), respectively). The experimental spectra were measured at a grazing incidence of θ = 20°. The lowest and 2nd lowest lying doublet features are assigned to transitions into the LUMO and LUMO+1, respectively. The vertical lines indicate the center position of the C K to LUMO and LUMO+1 transitions.

In Figure 5a, we compare the C 1s XAS spectra of **6A** films on Ag and Cu to a DFT simulation (top). The simulated spectra were obtained by broadening of the discrete excitations with Gaussian functions. This allows us to assign the observed spectral features to specific transitions and to disentangle contributions from inner C–C, inner C–H, and terminal C–H atoms analog to XPS (blue, black, red curves, respectively). Following earlier reports on **6A** (and also **5A**), the lowest lying main features in C 1s XAS spectra (photon energies <288 eV, denoted A–D in Figure 5), can be attributed to transitions into π* orbitals.^12,74,77,78^

This assignment is also supported by DFT calculations of the isolated molecule. Here, we assign the spectral features A and B in Figure 5 to be predominantly caused by transitions into the LUMO. The splitting of the LUMO transition into two main contributions reflects the different core level binding energies of the C atoms as described by XPS. Feature A is attributed to excitations from the inner C–H atoms, which show the lowest C 1s binding energy, while feature B originates from the terminal C–H atoms. The oscillator strength of transitions from the C–C atoms is minor as the LUMO is not directly localized on these atoms. The higher energy features C and D arise from transitions into succeeding π* orbitals, i.e., LUMO+1,2,3.

The two bottom spectra of Figure 5a are the experimental C 1s XAS of **6A** for multilayer thicknesses of 3.6 nm (Ag) and 9 nm (Cu). The spectra were taken at grazing incidence (20°), where the intensity for transitions into π* orbitals is maximal for flat lying molecules with a π-conjugated carbon system. The experimental spectra for the multilayer films in Figure 5a are in good agreement with both the literature^11^ and the simulations. Therefore, we assign the two features *A* and *B* (located at 283.6/284.3 eV) to transitions into the LUMO and the features *C* and *D* (285.6/286.2 eV) to transitions into the LUMO+1 and other orbitals of higher energies. We note that their relative intensities in the case of multilayer coverages in Figure 5a depend obviously on the underlying substrate, indicating a different arrangement of **6A** molecules. This might be plausible due to the different adsorption geometry of the first molecular layer on both substrates (cf. Figure 1). Polarization-dependent XAS spectra for multilayer films are shown in Figure S10 (Supporting Information), revealing a pronounced angular dependence of C 1s−π* transitions on Ag(110).

For the monolayers of **6A**/Ag and **6A**/Cu, the XAS spectral shapes are distinctly different compared to the corresponding multilayer spectra (Figure 5b) and even to each other. For **6A** on Ag(110), the peaks *A* and *B* previously assigned to the C 1s–LUMO transition have disappeared, and the higher energy region appears to be slightly modified. This can be rationalized by the population of the LUMO due to charge transfer, in good agreement with the results of POT and XPS. Regarding the changes around *C* and *D*, it should be noted that also the C 1s XPS signal was altered by the substrate-molecule interaction. For **6A**/Cu(110), not only the features *A* and *B* have completely vanished but also the intensity attributed to *C/D* is distinctly suppressed. This can be interpreted as population of the LUMO and at least partial population of the LUMO+1, in excellent agreement to complementary POT and XPS measurements.

## 4 Conclusion

The geometric structure and electronic properties of hexacene on Ag(110) and Cu(110) were studied using XPS, POT, XAS, STM, LEED, and DFT calculations. Similar to tetracene, hexacene adsorbs in two mirror domains on Ag(110), where the molecules are slightly rotated with respect to the [11̅0]-direction of the substrate. Differences of the adsorption geometry can be essentially ascribed to the length of the molecule. For **6A** on Cu(110), large single domains were observed, in which the long axis of the molecules is oriented parallel to the [11̅0]-direction of the substrate. The different behavior on both substrate surfaces can be understood by different lateral distances of the metal atom rows on Cu(110) and Ag(110) surfaces. XAS, XPS, and POT reveal a charge transfer from the metal substrates to the molecules. While only the LUMO is occupied for **6A**/Ag(110), also the LUMO+1 is at least partially filled in the case of **6A**/Cu(110). Theory suggests that the strength of the chemisorption has consequences for the adsorption height of the **6A** molecule on the respective substrate surface and concomitant on the resulting interface dipole. The results indicate that the detailed geometric structure of the substrate surface determines to a large extent the molecular orientation and thus also electronic interface properties. The experimental and theoretical study of hexacene’s structural and electronic properties on Ag and Cu presented here are supposed to instigate more in-depth analysis of adsorption of even longer acenes to be synthesized in future.

## Supporting Information Available

STM images of a **6A** adlayers on Ag(110), STM and LEED images of a **7A** adlayer on Ag(110), LEED images of **6A** monolayers on Cu(110) and Ag(110), valence band spectra of **6A** monolayers on Ag(110) and Cu(110), real space orbitals of the free hexacene molecule and the corresponding calculated momentum maps, peak fit parameters of the C 1s spectra of **6A** on Cu(110) and Ag(110), calculated vertical substrate–molecule distances, lateral view on the interfaces, charge density difference of the molecule–surface interfaces, and density of states of the freestanding monolayers, and XAS spectra of multilayer films of **6A** on Ag(110) and Cu(110) (PDF)
