# Supplementary material for: Hexacene on Cu(110) and Ag(110): Influence of the Substrate on Molecular Orientation and Interfacial Charge Transfer
Source: J Phys Chem C Nanomater Interfaces. 2022 Mar 7;126(10):5036–45. doi: 10.1021/acs.jpcc.2c00081 (PMC8935373; doi:10.1021/acs.jpcc.2c00081)
Supplement: Supplementary file 1 — jp2c00081_si_001.pdf [file jp2c00081_si_001.pdf]

Supporting Information

Hexacene on Cu(110) and Ag(110): Influence of the Substrate on Molecular Orientation and Interfacial Charge Transfer

*Marie S. Sättele,[1,2] Andreas Windischbacher,[3] Katharina Greulich,[1] Larissa Egger,[3] Anja Haags,[4,5,6] Hans Kirschner,[7] Ruslan Ovsyannikov,[8] Erika Giangrisostomi,[8] Alexander Gottwald,[7] Mathias Richter,[7] Serguei Soubatch,[4,5] F. Stefan Tautz,[4,5,6] Michael G. Ramsey,[3] Peter Puschnig,[3] Georg Koller,[3] Holger F. Bettinger,[2] Thomas Chassé,[1,9] Heiko Peisert[1]**

[1] Institute of Physical and Theoretical Chemistry, University of Tübingen, Auf der Morgenstelle 18, 72076 Tübingen, Germany

[2] Institute of Organic Chemistry, University of Tübingen, Auf der Morgenstelle 18, 72076 Tübingen, Germany

[3] Institute of Physics, University of Graz, NAWI Graz, Universitätsplatz 5, 8010 Graz, Austria

[4] Peter Grünberg Institut (PGI-3), Forschungszentrum Jülich, 52425 Jülich

[5] Jülich Aachen Research Alliance (JARA), Fundamentals of Future Information Technology, 52425 Jülich, Germany

[6] Experimental Physics IV A, RWTH Aachen University, 52074 Aachen, Germany

[7] Physikalisch-Technische Bundesanstalt, Abbestr. 2-12, 10587 Berlin.

[8] Institute for Methods and Instrumentation in Synchrotron Radiation Research, Helmholtz-Zentrum Berlin für Materialien und Energie GmbH, Albert-Einstein-Straße 15, 12489 Berlin, Germany

[9] Center for Light-Matter Interaction, Sensors & Analytics (LISA+) at the University of Tübingen, Auf der Morgenstelle 18, 72076 Tübingen, Germany

*Corresponding author, Heiko Peisert, heiko.peisert@uni-tuebingen.de, Tel.: (+49) 07071 / 29-76931, Fax: (+49) 07071 / 29-5490

STM images of 6A adlayer on Ag(110)

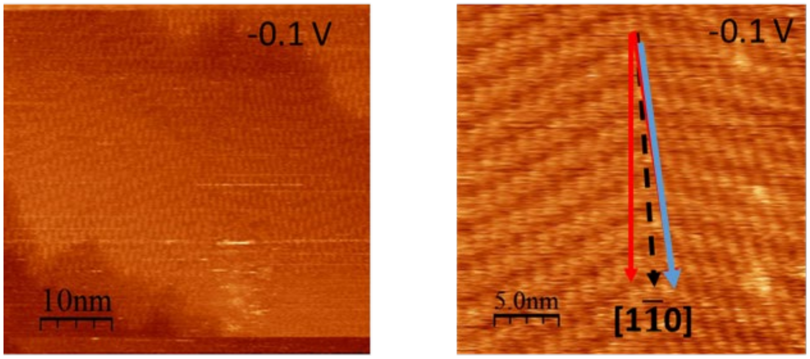

Figure S1 *6A on Ag(110): STM images of a 6A adlayer (a) 50x44 nm^2 (I =- 580 pA, V= -0.1 V) b) 20x18 nm^2 (-580 pA/ -0.1 V)*

STM and LEED images of a 7A adlayer on Ag(110)

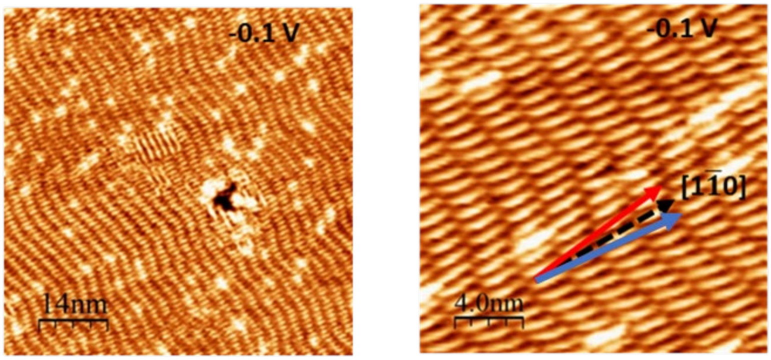

Figure S2 *7A on Ag(110): STM images of a 7A adlayer (a) (I =- 320 pA, V= -0.1 V) b) 20x18 nm^2 (-300 pA/ -0.1 V)*

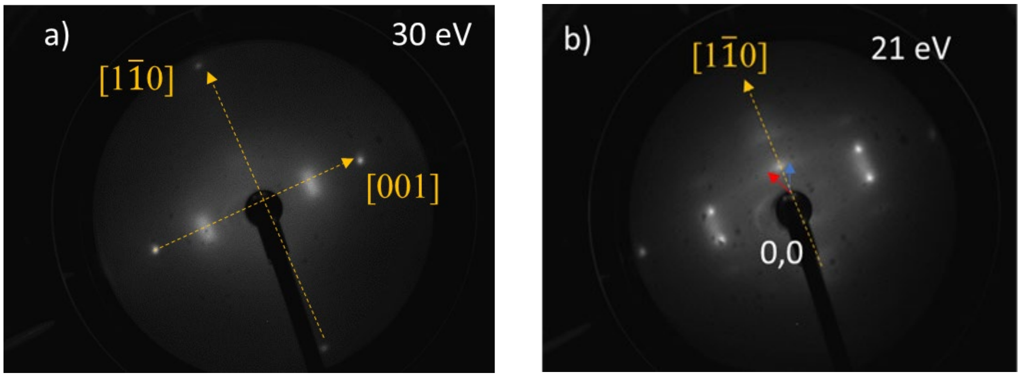

Figure S3: *LEED images of 7A monolayers on Ag(110) (a, b) recorded at different excitation energies.*

LEED images of 6A monolayers on Cu(110) and Ag(110)

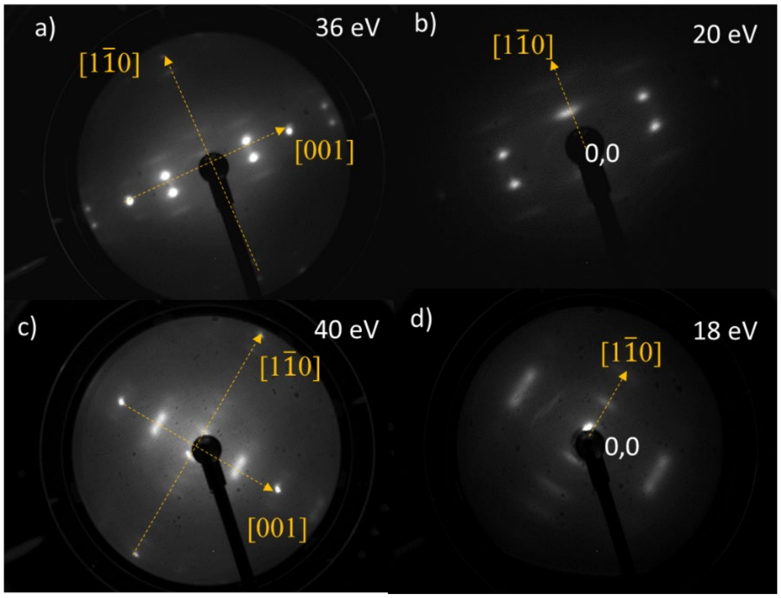

Figure S4: *LEED images of 6A monolayers on Ag(110) (a, b) und Cu(110) (c, d) recorded at different excitation energies.*

Valence band spectra of 6A monolayers on Ag(110) and Cu(110)

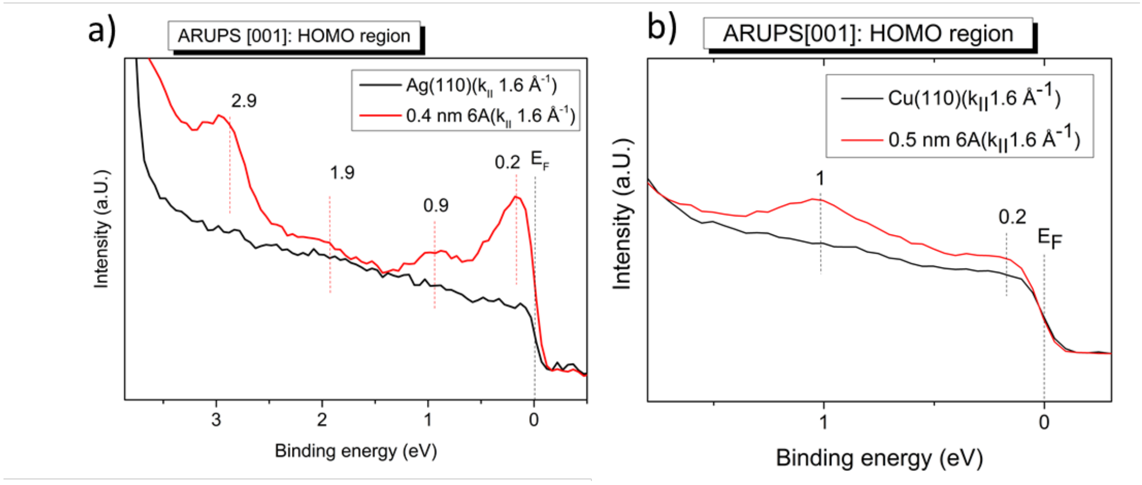

Figure S5: *ARPES measurements of a 6A monolayers on Ag(110) (**a**) and Cu(110) (**b**).*

Peak fit parameters of the C1s spectra of 6A on Cu(110) and Ag(110)

Peak fit parameters of the C1s spectra of a 6A multilayer and 6A monolayers on Cu(110) and Ag(110) with different carbon species are shown in **Table S1-S3**. C1s core level spectra were taken at hv = 1486.6 eV (pass energy 10 eV, step size 0.03 eV). The red component represents the outer, the black component the inner C-H carbon atoms, while the blue component are assigned to carbon atoms not being bonded to hydrogen atoms.

Table S1-S3: *Peak fit parameters of the C1s multilayer film and the monolayers on Cu(110) and Ag(110).*

Multilayer	***Peak height/ counts***	***Position/ eV***	***FWHM/ eV***	***Asym.***	***Rel. Area %***	***GP-FWHM***	***LP-FWHM***
red comp.	*11884*	*284.73*	*0.988*	*0*	*31*	*0.92*	*0.12*
black comp.	*11884*	*284.33*	*0.988*	*0*	*31*	*0.92*	*0.12*
blue comp.	*14855*	*284.96*	*0.988*	*0*	*38*	*0.92*	*0.12*

ML/Cu	***Peak height/ counts***	***Position/ eV***	***FWHM/ eV***	***Asym.***	***Rel. Area %***	***GP-FWHM***	***LP-FWHM***
red comp.	*3740*	*284.48*	*0.949*	*0.21*	*31*	*0.71*	*0.11*
black comp.	*3740*	*284.50*	*0.949*	*0.21*	*31*	*0.71*	*0.11*
blue comp.	*4675*	*285.05*	*0.949*	*0.21*	*38*	*0.71*	*0.11*

ML/Ag	***Peak height/ counts***	***Position/ eV***	***FWHM/ eV***	***Asym.***	***Rel. Area %***	***GP-FWHM***	***LP-FWHM***
black comp.	*7615*	*283.67*	*0.847*	*0.21*	*31*	*0.62*	*0.11*
red comp.	*7615*	*284.26*	*0.847*	*0.21*	*31*	*0.62*	*0.11*
blue comp.	*9519*	*284.5*	*0.847*	*0.21*	*38*	*0.62*	*0.11*

Calculated maps of the isolated molecule

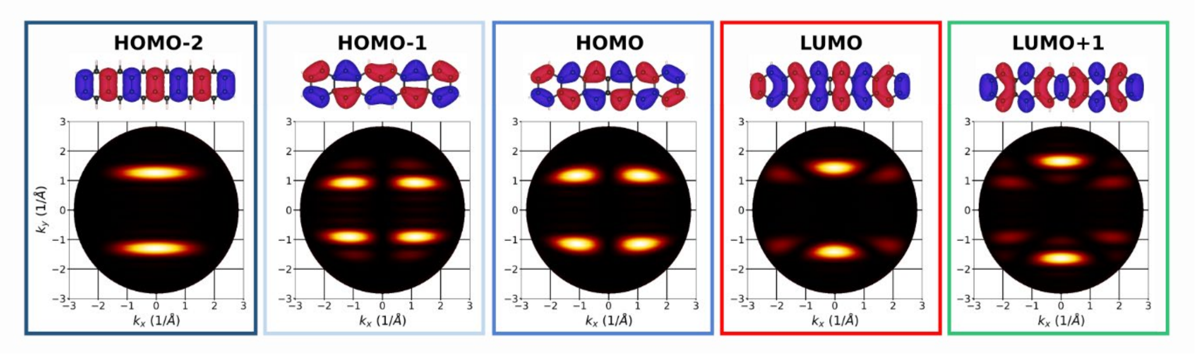

Figure S6: *Real space orbitals of the free hexacene molecule and the corresponding calculated momentum maps.*

Vertical substrate-molecule distances, lateral view on the interfaces, charge density difference of the molecule-surface interface and density of states of the freestanding monolayers

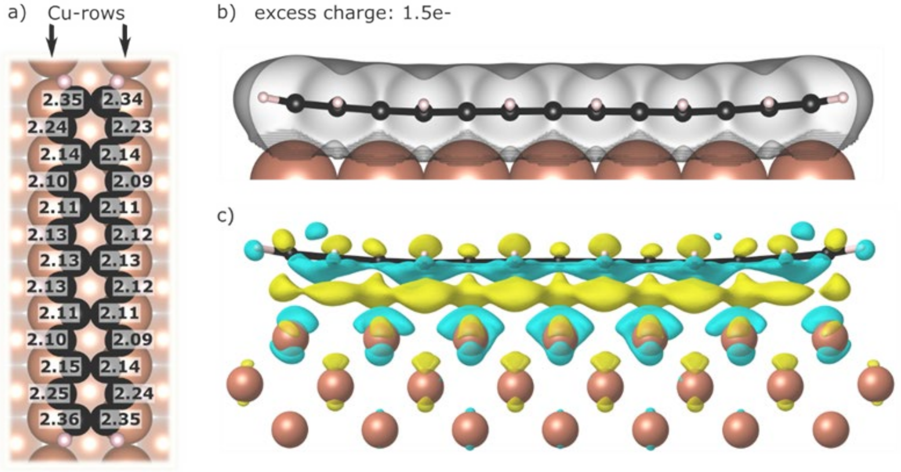

Figure S7: *Calculation details for the 6A/Cu(110) interface a) Simulated vertical substrate-molecule distance (Å), b) Lateral view of the interface including the volume, which was assigned to the molecule by Bader partitioning. Excess electron charge of this volume compared to the free molecule is given, c) Charge density difference of the molecule-substrate interface and its subsystems, yellow (blue) denotes gain (loss) in electron density upon adsorption, isovalue=0.002.*

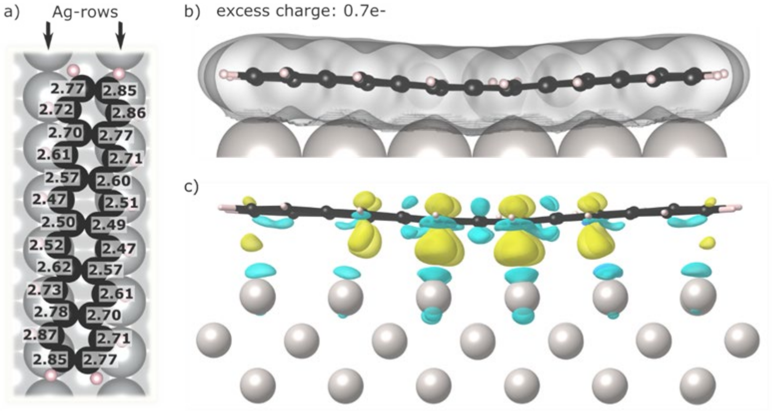

Figure S8: *Calculation details for the 6A/Ag(110) interface a) Simulated vertical substrate-molecule distance (Å), b) Lateral view of the interface including the volume, which was assigned*

to the molecule by Bader partitioning. Excess electron charge of this volume compared to the free molecule is given, c) Charge density difference of the molecule-substrate interface and its subsystems, yellow (blue) denotes gain (loss) in electron density upon adsorption, isovalue=0.002.

The **Figure S7,S8** shows the simulated vertical substrate-molecule distances for 6A in the hollow position along $[1\bar{1}0]$ with a rotation of 0° and 6° on Cu(110) and Ag(110). We investigated a bending of the molecules on both substrates, as the distance between the metal and carbon atoms in the middle of the 6A molecules is about 0.2-0.3 Å reduced as compared to the outer metal-carbon distances. On both substrates the distances are significantly lower as the sum of the van-der Waals-radii of C-Cu (3.1 Å) and C-Ag (3.41 Å).

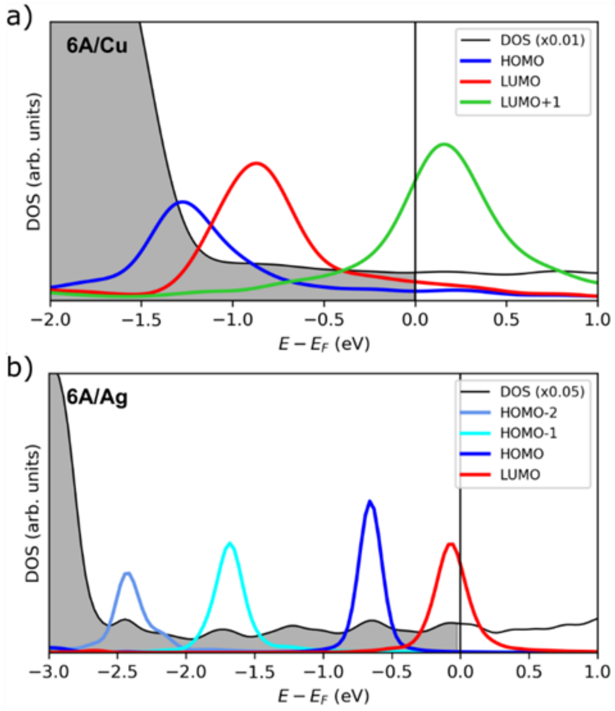

Figure S9: *Density of states of the full systems and projected onto the molecular orbitals of a freestanding monolayer. The full DOS is scaled by the given factor for better visualization. Note*

*that the simulated momentum maps of the interface shown in **Fig.2** were calculated at those energies corresponding to the respective maxima of the orbital projected DOS*

C K-edges of 6A multilayer films

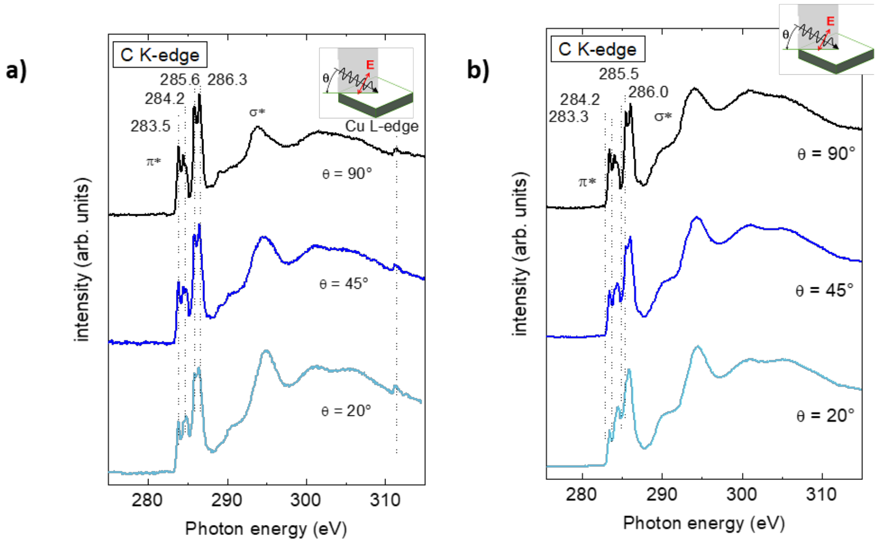

***Figure S10:** C K-edge of a multilayer film of 6A on Cu(110) (a) and Ag(110) (b).*

On both surfaces the spectra exhibit a very weak dichroism; some more anisotropy might be visible in the film on Ag(110) (**Figure S10**). This point out a preferred orientation (i.e. almost disordered molecules) or other reasons for example the molecules are oriented in a herringbone structure. Then we would not expect a strong dichroism if the polar angle was varied in the direction of the herringbone structure.
